# High level of EZH2 expression is linked to high density of CD8-positive T-lymphocytes and an aggressive phenotype in renal cell carcinoma

**DOI:** 10.1007/s00345-020-03200-4

**Published:** 2020-04-17

**Authors:** Till Eichenauer, Luca Simmendinger, Christoph Fraune, Tim Mandelkow, Niclas C. Blessin, Martina Kluth, Claudia Hube-Magg, Katharina Möller, Till Clauditz, Sören Weidemann, Roland Dahlem, Margit Fisch, Silke Riechardt, Ronald Simon, Guido Sauter, Franziska Büscheck, Michael Rink

**Affiliations:** 1 Department of Urology, University Medical Center Hamburg-Eppendorf, Hamburg, Germany; 2 Institute of Pathology, University Medical Center Hamburg-Eppendorf, Martinistraße 52, 20246 Hamburg, Germany

**Keywords:** Renal cell carcinoma, CD8, EZH2, Tissue microarrays

## Abstract

**Purpose:**

Enhancer of zeste homolog 2 (EZH2), the catalytic part of the Polycomb repressive complex 2 (PRC2), has a prognostic role in renal cell carcinoma (RCC) and was recently shown to modulate the immune response by reducing tumor cell immunogenicity.

**Methods:**

To investigate whether the prognostic role of EZH2 might be driven by a modified immune environment, more than 1800 RCCs were analyzed in a tissue microarray for EZH2 expression and CD8 positive lymphocytes were quantitated by automated digital imaging.

**Results:**

EZH2 positivity was found in 75.2% of 1603 interpretable tumors. In clear cell RCC, high EZH2 expression was significantly linked to high ISUP, Furmann, and Thoenes grade (*p* < 0.0001 each), advanced stage (*p* < 0.0001), nodal (*p* = 0.0190) and distant metastasis (*p* < 0.0001) as well as shortened overall (*p* < 0.0027) and recurrence free survival (*p* < 0.0001). The density of CD8+ cells varied from 0 to 5048 cells/mm^2^ (Median 120 cells/mm^2^). A high CD8+ count was significantly associated with high ISUP, Fuhrmann, and Thoenes grade (*p* < 0.0001 each), advanced tumor stage (*p* = 0.0041), distant metastasis (*p* = 0.0026) as well as reduced overall survival (*p* = 0.0373) and recurrence free survival (*p* = 0.0450). The density of CD8+ cells continuously increased with raising EZH2 levels (*p* < 0.0001).

**Conclusion:**

Our data support a striking prognostic role of both EZH2 expression and the density of CD8+ cells in RCC. The tight relationship of EZH2 expression and CD8+ cell counts in RCC is consistent with models suggesting that EZH2 overexpression can be caused by high lymphocyte content in certain tumor types. Such a mechanism could explain the unique finding of high lymphocyte counts driving poor prognosis in RCC patients.

## Introduction

Renal cell carcinoma (RCC) is one of the most common tumors worldwide [1]. Localized tumors are generally treated surgically, whereas for advanced tumors necessitating systemic treatment, several new drugs were approved lately and have improved the still unfavorable prognosis of metastatic disease [2, 3]. As in other cancer types, immune-checkpoint inhibitors are in focus of current kidney cancer research [4–6]. In clear cell RCC, a combination of the Ipilimumab (CTLA-4 inhibitor) and nivolumab (PD-1 inhibitor), pembrolizumab (PD-1 inhibitor) and axitinib (VEGFR inhibitor), and avelumab (PD-L1 inhibitor) and axitinib (VEGFR inhibitor) showed superior survival in a phase III study and is now recommended as first line systemic therapy in intermediate- and poor-risk patients [2, 7–9].

Current clinical trials evaluate whether adjuvant application of immune-checkpoint inhibitors or other new drugs can improve the prognosis of patients with kidney cancer in high risk situations, such as tumor recurrence or progression after nephrectomy (Keynote-564, IMmotion010, Checkmate-914). If adjuvant treatment becomes standard of care, risk stratification will become more important than ever before, to find out which patient is at risk and might benefit from extended treatment. A better understanding of disease biology will potentially lead to the identification of clinically applicable molecular markers that enable a more reliable prediction of kidney cancer aggressiveness.

Enhancer of zeste homolog 2 (EZH2) is of potential interest in kidney cancer. EZH2 is the catalytic part of the Polycomb repressive complex 2 (PRC2) [10]. Through trimethylation of Histone 3 on lysine 27 (H3K27me3) it induces chromatin compaction and transcriptional repression of various genes including p16 and E-Cadherin [11, 12]. EZH2 also interacts with various non-histone proteins and can serve as a transcriptional activator [13]. Studies have suggested that high levels of EZH2 expression in cancer tissue may be strongly linked to poor patient prognosis. This includes several studies on kidney cancer [14–22]. Importantly, EZH2 has recently been shown to modulate the immune response to tumor cells by reducing their immunogenicity [23].

To investigate whether the prognostic role of EZH2 might be driven by a modified immune response, a cohort of 1809 renal cell carcinomas was analyzed in a tissue microarray format (TMA) for EZH2 protein expression and CD8+ lymphocytes were quantitated by automated digital imaging.

## Materials and methods

### Patients

A set of kidney tumor tissue microarrays (TMAs) was used containing one tissue core each from 1809 kidney tumors routinely diagnosed from nephrectomy specimen between 1994 and 2016 at the Institute of Pathology of the University Medical Center Hamburg-Eppendorf, Germany. All tumors had been reviewed according to the criteria described in the 2016 WHO classification by two pathologists with a special focus on genitourinary pathology (FB, CF) and ISUP (International Society of Urologic Pathologists) grading was performed for each tumor. Follow-up data were available for 777 of 1176 clear cell cancers. Available study endpoint were overall survival and recurrence free survival, including patients without metastasis (M0) at the timepoint of surgery and patients with initial metastasis (M1) and additional progress after surgery. The TMA comprises four blocks, one of which had been earlier used [24]. The TMA manufacturing process was described in detail before [25]. In brief, from each donor tumor, one tissue core measuring 0.6 mm in diameter was taken from a tumor-containing tissue block. Clinical and pathological parameters of the arrayed tumors are summarized in Table 1. The mean follow-up time was 48 months.

**Table 1:** Clinico-pathological features of 1809 arrayed renal cell cancers

	Study cohort on TMA (*n* = 1809)
Follow-up	
Available (*n*)	1174
Mean (months)	48
Median (months)	61.8
Age (years)	
< 50	263
50–70	951
70–90	595
Histology	
Clear cell RCC	1167
Papillary RCC	270
Chromophobe RCC	101
Oncocytoma	149
UICC stage	
I	733
II	131
III	175
IV	158
pT category	
pT1	998
pT2	223
pT3–4	408
ISUP grade	
1	398
2	537
3	469
4	100
Fuhrman grade	
1	72
2	851
3	480
4	110
Thoenes grade	
1	497
2	839
3	177
pN category	
pN0	232
PN+	49
pM category	
pM0	220
pM+	148

Numbers do not always add up to 1809 in the different categories because of missing data

### Immunohistochemistry (IHC)

Freshly cut TMA sections were immunostained on 1 day and in one experiment. Slides were deparaffinized and exposed to heat-induced antigen retrieval for 5 min in an autoclave at 121 °C at pH 9 (EZH2) or pH 7.8 (CD8) Tris–EDTA-Citrate buffer. Primary antibody specific for EZH2 (mouse monoclonal antibody, Abnova, Taipeh, Taiwan; cat#MAB9542; dilution 1:150) was applied at 37 °C for 60 min. Bound antibody was then visualized using the EnVision Kit (Dako, Glostrup, Denmark) according to the manufacturer’s directions. EZH2 staining was predominantly nuclear and no staining was found in normal tissue. EZH2 staining was typically found in either all (100%) or none (0%) of the tumor cells in a given cancer spot. Staining intensity of all cases was thus semi-quantitatively assessed in four categories: negative, weak, moderate and strong. The percentage of positive tumor cells (typically 100%) was not separately recorded. For CD8 staining, the slides were deparaffinized, rehydrated, exposed to heat-induced antigen retrieval for 15 min at 98 °C in pH9 DAKO target retrieval Solution (S2367) using a DAKO PT-LINK device and then transferred to a DAKO Link 48 autostainer device. The autostainer protocol includes peroxidase blocking for 5 min (DAKO, Envision Flex-Kit 8002) and subsequent incubations of the primary antibody (Oncodianova, mouse monoclonal antibody, Clone TC8, dilution 1:200) for 20 min at room temperature, Flex HRP (DAKO EnVision Flex-Kit 8002) for 20 min, DAB-Chromogen (DAKO EnVision Fley-Kit 8002) for 10 min as well as a final incubation with Hämatoxylin (DAKO K8008) for 5 min.

### Quantification of CD8 immunostaining

Digital images of CD8-stained slides were acquired using Leica’s Aperio VERSA 8 automated microscope. TMA spots were automatically identified and analyzed using Image Scope 12.3.3 software package (Leica Microsystems, Wetzlar, Germany) according to the following procedure: Every TMA slide was scanned at 40× magnification. Digital images were segmented using the Image Scope TMA-Tool and the segmentation was corrected manually. Two Aperio ePathology Image Analysis macros were adjusted (Leica Microsystems, Wetzlar, Germany) to determine the number of CD8+ cells in each tissue spot and to measure the corresponding exact area of each tissue spot. The number of stained cells and the area in square millimeters of each individual spot was used to calculate the density of stained cells/mm^2^ (number of cells per square mm). Threshold selection was done according to Galon et al. [26]. In brief, the cutoff providing the highest survival difference was selected. In our study, this cutoff was 142 cells/mm^2^ to separate “low” from “high” CD8 density.

### Statistics

Statistical calculations were performed with JPM 14 software (SAS Institute Inc, NC, USA). Contingency tables and the Chi-square test were performed to search for associations between EZH2 or CD8 and tumor phenotype as well as tumor subtype. Survival curves were calculated according to Kaplan–Meier. The log-rank test was applied to detect significant survival differences between groups analysis of variance (ANOVA) tests were used to investigate the relationship between categorical and continuous data.

### Technical issues

1603 of 1809 (88.6%) tissue spots were informative for EZH2. Reasons for non-informative TMA spots were lack of tissue spots in the TMA section or lack of sufficient amounts of tumor cells in a TMA spot. For CD8, only an “unusual” low number of tissue spots (1163 of 1809, 64.3%) were interpretable, because tumors spots lacking detectable CD8+ lymphocytes were excluded from further analysis. Accordingly, analysis of both markers (CD8 and EZH2) was successful in 1,075 of 1,809 (59.4%) of the arrayed cancers.

## Results

### EZH2 expression in renal cell cancer

**Fig. 1:**
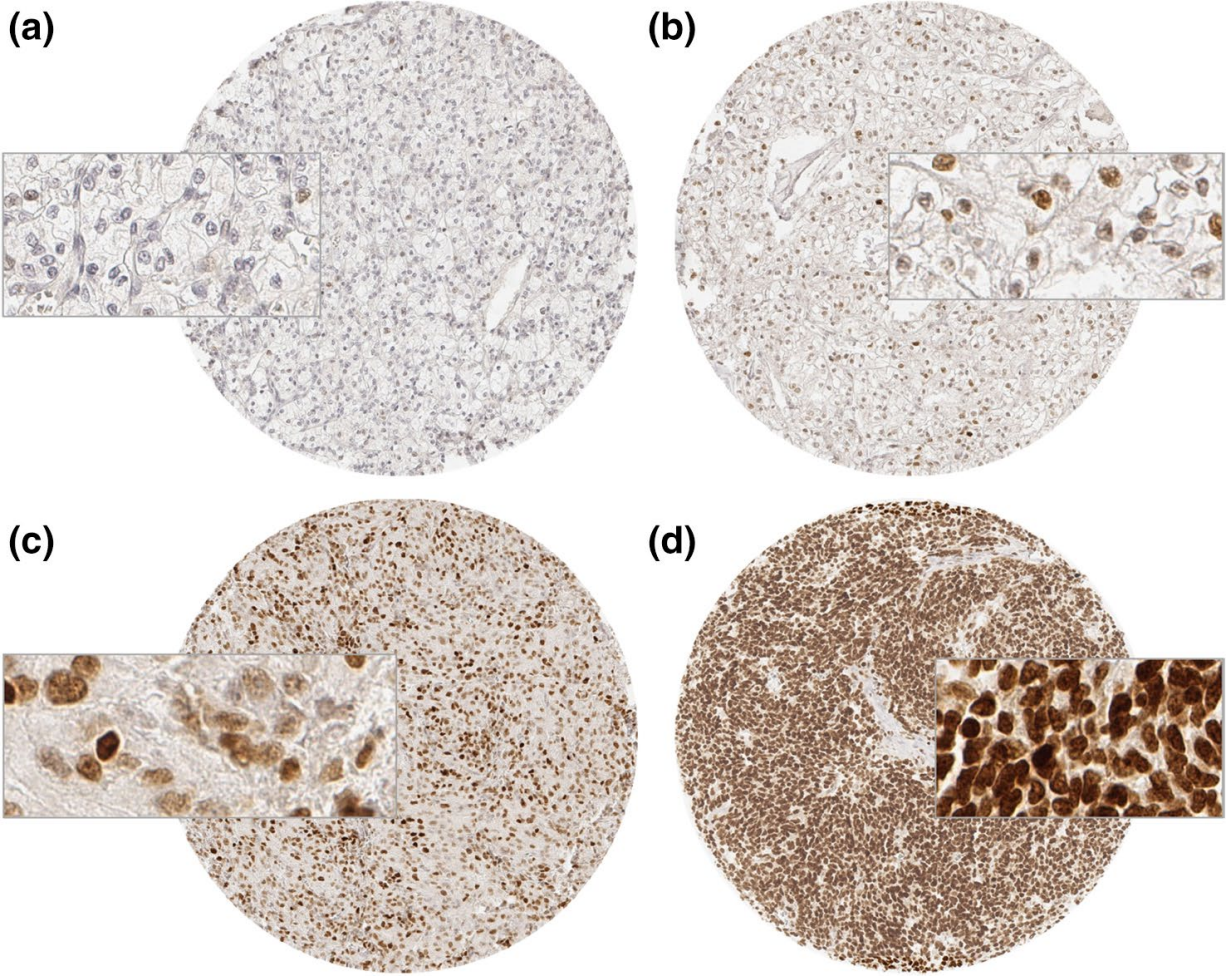
EZH2 immunostaining in clear cell renal cell carcinomas. **a** Negative staining, **b** weak staining, **c** moderate staining, and **d** strong staining

**Fig. 2:**
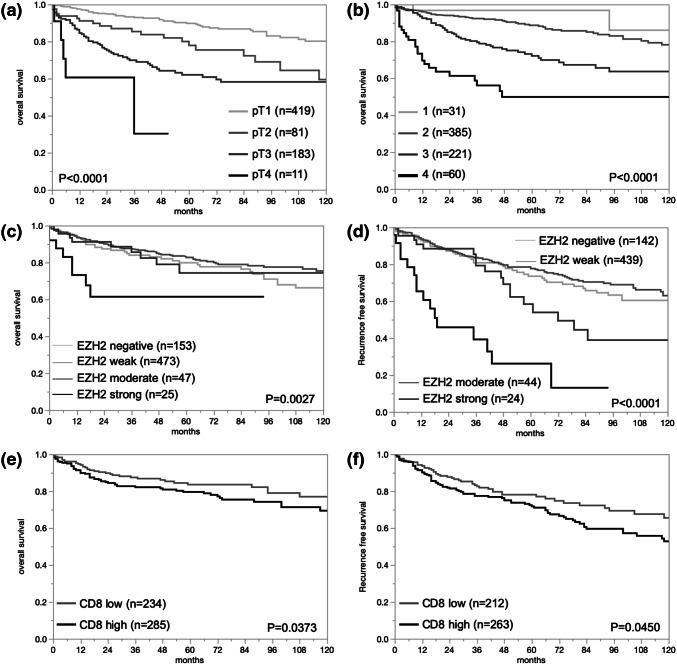
Comparison to prognosis in renal cell carcinomas (RCC). **a** pT and overall survival in clear cell RCC (ccRCC), **b** Fuhrmann grading and overall survival in ccRCC, **c** EZH2 immunostaining and overall survival in ccRCC, **d** EZH2 and recurrence free survival in ccRCC, **e** CD8 and overall survival in ccRCC, and **f** CD8 and recurrence free survival in ccRCC

In normal kidney tissue EZH2 staining was not observed, but in a fraction of cancers, nuclear EZH2 immunostaining was present, however. Representative images of EZH2 positive and negative cancers are given in Fig. 1. In total, EZH2 positivity was found in 75.2% of 1603 analyzable cancers, including 63.1% tumors with weak, 8.0% with moderate, and 4.1% with strong immunostaining according to our criteria. EZH2 expression varied slightly between the different histological subtypes (Table 2). EZH2 expression was most commonly seen in papillary cancers (87.0% positive) and least frequent in oncocytomas (63.1%) and chromophobe cancers (64.1%; Table 2). The search for associations between EZH2 expression and tumor phenotype was limited to the largest subset of 1053 analyzable clear cell RCC (Table 3). The number of samples was too small for a statistically meaningful analysis in papillary, chromophobe and other cancer types. In clear cell RCC, high (moderate to strong) EZH2 expression was significantly linked to high ISUP, Fuhrmann, and Thoenes grade (*p* < 0.0001 each), advanced stage (*p* < 0.0001), nodal metastasis (*p* = 0.0190), and distant metastasis (*p* < 0.0001). Follow-up data were available for 698 (overall survival) or 649 (recurrence free survival) clear cell cancers with analyzable EZH2 immunostaining. The validity of the follow-up data is demonstrated in Fig. 2a, b, where significant associations between tumor stage or Fuhrman grade with overall survival are shown in the subset of clear cell cancers. There was a statistically significant association between high EZH2 expression and reduced overall survival (*p* = 0.0027, Fig. 2c) and recurrence free survival in the subset of clear cell RCC (*p* < 0.0001, Fig. 2d).

**Table 2:** Comparison between EZH2 immunostaining and renal cell cancer subtypes

Entity	Analyzable (*n*)	EZH2			
		Negative (%)	Weak (%)	Moderate (%)	Strong (%)
Renal cell cancers (RCC)	1603	397 (24.8%)	1012 (63.1%)	128 (8%)	66 (4.1%)
Clear cell RCC	1053	266 (25.2%)	691 (65.6%)	63 (6%)	34 (3.2%)
Papillary RCC	238	31 (13%)	144 (60.5%)	49 (20.6%)	14 (5.9%)
Chromophobe RCC	92	33 (35.9%)	54 (58.7%)	4 (4.3%)	1 (1.1%)
Clear cell tubulopapillary RCC	28	7 (25%)	19 (67.9%)	2 (7.1%)	0 (0%)
Oncocytomas	122	45 (36.9%)	74 (60.7%)	2 (1.6%)	1 (0.8%)
Other rare types	69	15 (21.7%)	30 (43.5%)	8 (11.6%)	16 (23.2%)

**Table 3:** Comparison of EZH2 immunostaining and clinico-pathological features in clear cell

Parameter	Analyzable (*n*)	Negative (%)	Weak (%)	Moderate (%)	Strong (%)	*p* value
ISUP						
1	275	86 (31.3%)	182 (66.2%)	5 (1.8%)	2 (0.7%)	< 0.0001
2	353	106 (30%)	232 (65.7%)	12 (3.4%)	3 (0.9%)	
3	338	60 (17.8%)	234 (69.2%)	29 (8.6%)	15 (4.4%)	
4	78	13 (16.7%)	35 (44.9%)	16 (20.5%)	14 (18%)	
Fuhrmann						
1	42	15 (35.7%)	27 (64.3%)	0 (0%)	0 (0%)	< 0.0001
2	574	173 (30.1%)	381 (66.4%)	15 (2.6%)	5 (0.9%)	
3	348	62 (17.8%)	239 (68.7%)	34 (9.8%)	13 (3.7%)	
4	89	16 (18%)	43 (48.3%)	14 (15.7%)	16 (18%)	
Thoenes						
1	342	106 (31%)	222 (64.9%)	11 (3.2%)	3 (0.9%)	< 0.0001
2	572	137 (24%)	390 (68.2%)	32 (5.6%)	13 (2.3%)	
3	139	23 (16.6%)	78 (56.1%)	20 (14.4%)	18 (13%)	
Tumor stage						
pT1	593	145 (24.5%)	415 (70%)	25 (4.2%)	8 (1.4%)	< 0.0001
pT2	134	38 (28.4%)	85 (63.4%)	7 (5.2%)	4 (3%)	
pT3	307	79 (25.7%)	182 (59.3%)	27 (8.8%)	19 (6.2%)	
pT4	13	2 (15.4%)	4 (30.8%)	4 (30.8%)	3 (23.1%)	
Lymph node metastasis						
pN0	164	37 (22.6%)	113 (68.9%)	10 (6.1%)	4 (2.4%)	0.019
pN1	10	1 (10%)	6 (60%)	1 (10%)	2 (20%)	
pN2	22	3 (13.6%)	11 (50%)	6 (27.3%)	2 (9.1%)	
Distant metastasis						
pM0	138	31 (22.5%)	102 (73.9%)	5 (3.6%)	0 (0%)	< 0.0001
pM1	119	30 (25.2%)	64 (53.8%)	12 (10.1%)	13 (10.9%)	

Numbers do not always add up to 1053 in the different categories because of missing data renal cell cancers

### Density of CD8+ T-lymphocytes in renal cell carcinoma

The count of CD8+ cells differed widely from 0 to 5048 cells/mm^2^ in 1163 interpretable cancers (Median 120; mean 342 cells/mm^2^). The density of CD8+ cells differed markedly between different tumor entities (Table 4) and was lowest in oncocytomas (mean 47.2) and highest in clear cell carcinomas (430.4). The comparison of the density of CD8+ cells with tumor phenotype and outcome was again limited to 770 clear cell RCC. A high density of CD8+ cells was significantly associated with high ISUP, Fuhrmann, and Thoenes grade (*p* < 0.0001 each) and distant metastasis (*p* = 0.0026, Table 5). High numbers of CD8, defined as ≥ 142 cells/mm^2^, were also linked with reduced overall survival (*p* = 0.0373) and recurrence free survival (*p* = 0.0450, Fig. 2e, f).

**Table 4:** Comparison between the density of CD8+ cells and renal cell cancer subtypes

Entity	Density of CD8+ cells (cell/mm^2^)		
	Analyzable (*n*)	Mean	SD
Renal cell cancers (RCC)	1163	342.3	611.7
Clear cell RCC	770	430.4	21.5
Papillary RCC	173	189.5	45.4
Chromophobe RCC	60	87.5	77.1
Clear cell tubulopapillary RCC	19	191.0	137.1
Oncocytomas	85	47.2	64.8
Other rare types	56	374.1	79.8

**Table 5:** Comparison of the density of CD8+ cells and clinico-pathological features in clear cell renal cell cancers

Parameter	Density of CD8+ cells (cell/mm^2^)			
	Analyzable (*n*)	mean	SD	*p* value
ISUP				
1	212	269.7	46.2	< 0.0001
2	255	378.8	42.2	
3	233	596.1	44.1	
4	61	576.5	86.2	
Fuhrmann				
1	41	210.2	105.1	< 0.0001
2	420	346.2	32.8	
3	240	564.0	43.5	
4	68	616.9	81.6	
Thoenes				
1	258	299.2	42.1	< 0.0001
2	408	467.6	33.4	
3	103	615.4	66.6	
Tumor stage				
pT1	448	374.1	31.9	0.0734
pT2	80	461.1	75.6	
pT3	225	517.5	45.1	
pT4	12	443.6	195.2	
Lymph node metastasis				
pN0	116	432.5	54.6	0.3965
pN1	7	132.5	222.4	
pN2	17	357.4	142.7	
Distant metastasis				
pM0	94	357.7	70.1	0.0383
pM1	85	570.0	73.7	

Numbers do not always add up to 770 in the different categories because of missing data

### Multivariate analyses

In clear cell cancers, multivariate analyses including the established prognosticators pT, pN, pM and Fuhrmann grade in addition to either EZH2 immunostaining or CD8+ cell counts showed that neither EZH2 nor the density of CD8+ cells were independent prognosticators (overall survival: *p* = 0.2970 for EZH2 and *p* = 0.1179 for CD8; recurrence free survival *p* = 0.3890 for EZH2 and *p* = 0.6002 for CD8).

### EZH2 expression and CD8 cell count

**Fig. 3:**
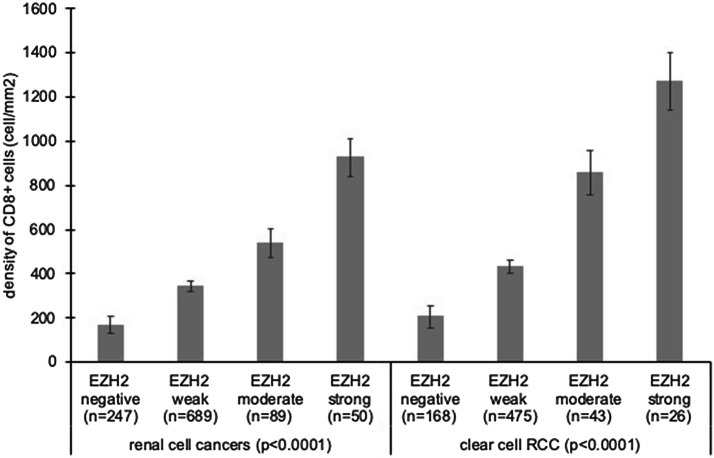
Association of EZH2 immunostaining and the density of CD8+ cells

Data on both EZH2 and CD8 immunohistochemistry was available for 1075 cancers. The comparison between EZH2 expression and CD8+ cell counts revealed that the number of CD8+ cells continuously increased with raising EZH2 levels. This held true for the group of all cancers (*p* < 0.0001) as well as for the subgroup of clear cell RCC (Fig. 3, *p* < 0.0001).

## Discussion

In this study EZH2 immunostaining was seen in 75.2% of kidney cancers. This is in the middle of the range reported in earlier studies. Using immunohistochemistry, EZH2 protein was detected in 44% of 110 [22], 78% of 165 [17], 84% of 119 [27], 89% of 520 [16], 100% of 257 [28], 100% of 185 [19], and 100% of 50 [29] kidney cancers. Differences in the EZH2 levels between these studies are likely to be caused by differences between antibodies selected, protocols used for immunostaining, and/or criteria for interpretation. Given the relatively mild differences between different kidney cancer subgroups observed in our study, it appears unlikely that the composition of RCC subtypes was a major contributing factor.

We do not consider it as a limitation that a TMA with only one 0.6 mm tissue core per tumor was analyzed in our study. Early studies have demonstrated, that all known associations between molecular features and cancer phenotype could be reproduced in TMAs. For example, Kononen et al. found the same frequencies of HER2, cMYC and Cyclin D1 in breast cancer as were expected in previous large sections studies [25]. Nocito et al. determined the Ki67LI on four replicated TMAs and associated large sections and found the same associations between Ki67LI and tumor phenotype and prognosis in both applications in bladder cancer [30]. Although it is possible that some positive EZH2 cancers were not detected because of potential genetic heterogeneity, we have previously shown that the usage of one tissue core per cancer is sufficient to find relevant associations between molecular alterations and tumor phenotype or patient prognosis [31, 32]. In an extensive analysis of multiple molecular markers in sets of 50–12,000 prostate cancers, we demonstrated the relationship between the number of analyzable tumors and the statistical power of TMA studies [33]. In a typical TMA analysis with one spot per cancer, about 60–80% of tumors will be analyzable. Obviously, this fraction can be increased if the number of samples per tumor is increased. However, considering that the analysis of 100 cancers with five spots each will result in 90–100 interpretable cancers, while a TMA made from 500 cancers with one spot each will result in 300–350 interpretable cancers, we strongly feel that analysis of one tissue core per cancer is preferable over the analysis of multiple cores.

The strong association between high EZH2 protein overexpression and poor patient outcome found in our cohort is well consistent with data from earlier studies. We did not analyze the mechanisms underlying EZH2 overexpression in our tumor cohort, but there is evidence from the literature that NF-kB, MUC1-C, KRAS, MEK-ERK and PI3K-AKT signaling might cause significant EZH2 up regulation in human cancers [34–36]. Interestingly, there are reports that infiltration of CD8+ lymphocytes may be among these factors at least in bladder, prostate and renal cell cancer [37–39]. Associations between high EZH2 levels and poor patient outcome in RCC patients has been described in nine earlier IHC-based reports [14–22]. In addition, an analysis of the RNA expression data of 1992 kidney tumors collected from several databases including The Cancer Genome Atlas (TCGA) suggested that high EZH2 expression might serve as a predictor for early cancer specific death in low and intermediate risk tumors, but not in high risk tumors [20]. All earlier studies had either analyzed clear cell RCC [15, 18–21] or cohorts containing various RCC subtypes [14, 16, 17, 22]. We used overall survival instead of cancer specific survival as an endpoint of our study for two reasons. First, tumor specific data were available only for a small subset of our patients. Second, it is difficult to judge whether such data actually reflect the reason for death in a generally elderly patient cohort.

For the purpose of this study, the CD8+ cytotoxic T-lymphocyte density was quantitated using a digital image analysis algorithm to calculate the number of CD8+ cells per measured square millimeter. The density of CD8+ cells obtained by this approach on our RCC TMA was comparable to the values that we earlier collected for clear cell RCC (306 ± 479; this study 430 ± 22) and papillary RCC (141 ± 149; this study 190 ± 45) in a study assessing the number of CD8+ cells across a broad range of different tumor entities (manuscript under revision, Cellular Oncology). The association between high density of CD8+ cells and poor patient outcome is consistent with data from two earlier studies, analyzing 221 [40] and 135 [40] RCCs. Overall, these findings describe an unusual role of CD8+ cells in RCC as high numbers of CD8+ cells are strongly linked to favorable prognosis in most other cancer types including advanced urothelial cancer [41], breast cancer [42], pancreatic carcinomas [43], colorectal cancer [44] and others. The link between high density of CD8+ cells and favorable prognosis is so strong in colorectal cancer, that various authors suggest lymphocyte quantification to become a routine procedure in these tumors [26, 45–47]. It has been suggested, that the different clinical impact of CD8+ cells between RCC and colorectal carcinoma may either reflect different tumor tissue organization or relate to tumor type specific other factors of the tumor microenvironment [40].

It is of note that the prognostic role of the density of CD8+ cells in RCC might be depending on the type of previous treatments, as recent work has suggested. George et al. analyzed the density of CD8+ cells in patients with locoregional high-risk RCC who were either treated with Sunitinib or Placebo in the S-TRAC trial and found that increased density of CD8+ cells was associated with longer disease free survival in the group of patients treated with Sunitinib, but not in the group treated with Placebo [48]. These authors suggested that cancer necrosis induced by VEGF/VEGFR inhibition and tumor hypoxia might result in an increased exposure of neoantigens that could be recognized and targeted by CD8+ cells. Necrosis-driven neoantigen exposure is a mechanism that may be unrelated to the type of therapy. However, Yao et al. [49] recently found that the role of CD8 might depend on the type of therapy, as patients with high density of CD8+ cells in metastases from RCCs only showed prolonged overall survival when sunitinib, but not sorafenib was the prior treatment.

The strong link between high EZH2 expression levels and a high density of CD8+ cells found in our study is consistent with a functionally relevant interaction of EZH2 protein expression and the immune response to tumor cells. This notion is supported by recent data of Zingg et al. [23] demonstrating that intratumoral T-cell accumulation—among other factors—can result in increased EZH2 expression in melanoma cells, which in return leads to a loss of immunogenicity and antigen presentation in EZH2 overexpressing tumor cells. EZH2 inactivation in this scenario caused a regain of immunogenicity. As similar findings could not be obtained in lung and colon cancer cells, the authors concluded that this mechanism might not be universally applicable but dependent on the tumor type [23]. Based on these observations, one might speculate that lymphocytic infiltration might cause a particular strong EZH2 overexpression in RCC and the resulting immune evasion causes the poor patient outcome in kidney cancers with high CD8+ cell density. A RCC specificity of such a mechanism could explain the unique association between high density of CD8+ cells and unfavorable disease outcome in RCC which markedly differs from findings in most other cancer types (i.e., urothelial cancer [41], breast cancer [42], pancreatic carcinomas [43], colorectal cancer [44] and others [50]).

In summary, our data show that high EZH2 protein expression is linked to poor prognosis in RCC. The tight relationship of EZH2 expression levels and the density of CD8+ cells in RCC is consistent with recent functional data by Zingg et al. [23] and might explain the unusual finding of a link between high lymphocyte content and poor prognosis in RCC patients.
